# Association between musculoskeletal disorders and health-related quality of life in Chilean and Spanish taxi drivers

**DOI:** 10.3934/publichealth.2026005

**Published:** 2026-01-07

**Authors:** Marta Marín-Berges, Alejandro Gómez-Bruton, Pablo A. Lizana, Maximiliano Cáceres, Pía Magaña, Isabel Iguacel

**Affiliations:** 1 IBiOPS, Instituto de Investigación Sanitaria Aragón, Universidad de Zaragoza, Zaragoza, Spain; 2 Centro de Investigación Biomédica en Red de Fisiopatología de la Obesidad y Nutrición (CIBEROBN), Instituto de Salud Carlos III, Madrid, Spain; 3 EXER-GENUD (Growth, Exercise, Nutrition and Development), Faculty of Health and Sport Sciences, Huesca, University of Zaragoza, Spain; 4 Laboratory of Epidemiology and Morphological Sciences, Instituto de Biología, Pontificia Universidad Católica de Valparaíso, Valparaíso 2373223, Chile; 5 Center for Interdisciplinary Research in Biomedicine, Biotechnology and Well-Being (CID3B), Pontificia Universidad Católica de Valparaíso, Valparaíso, Chile; 6 Instituto Agroalimentario de Aragón (IA2), Zaragoza, Spain; 7 NUTRI-GENUD (Growth, Exercise, Nutrition and Development), Faculty of Health Sciences, Zaragoza, University of Zaragoza, Spain

**Keywords:** musculoskeletal pain, quality of life, health-related quality of life, taxi driver, mental health

## Abstract

**Purpose:**

Taxi drivers are often exposed to long working hours, sedentary lifestyles, and prolonged static postures, increasing their risk of developing musculoskeletal disorders (MSDs) and negatively affecting their health-related quality of life (QoL). Robust estimates linking the burden of MSDs with QoL in taxi drivers remain scarce, especially in interregional comparisons. Therefore, we estimated the prevalence of MSDs and examined whether the number and distribution of painful regions were associated with QoL, adjusting for key sociodemographic factors, in samples of drivers from Chile and Spain.

**Methods:**

This cross-sectional study assessed 217 professional drivers (102 in Valparaíso, Chile, and 115 in Zaragoza, Spain) using validated questionnaires to assess MSD (Nordic Standardised Questionnaire), QoL (SF-36), and socio-demographic characteristics. Bivariate analyses and logistic regression models were applied.

**Results:**

A high prevalence of MSDs was reported (83.0%), with the lower back (51.6%), neck (50.2%), and right shoulder (34.5%) being the most affected regions. In both countries, the presence of two or more painful regions was significantly associated with worse physical QoL (Chile *OR* 5.8, 95% *CI* 2.20–15.27; Spain *OR* 3.4, 95% *CI* 1.39–8.22). Among Chilean drivers, older age and the perception of a precarious financial situation were statistically associated with poorer physical QoL (*OR* 1.1, 95% *CI* 1.01–1.09; *OR* 2.8, 95% *CI* 1.14–7.04, respectively). In Spain, the perception of financial difficulties was associated with poorer mental QoL (*OR* 3.1, 95% *CI* 1.08–8.84).

**Conclusion:**

These findings highlight the physical strain and vulnerability of professional drivers, underscoring the need for ergonomic initiatives and policy measures tailored to their context.

## 1. Introduction

Taxi driving is considered a high-risk occupation, not only due to environmental and job-related factors but also because of the irregular and often precarious working conditions involved [1]. While some taxi drivers may enjoy stable incomes and adequate working conditions, others face considerable challenges, such as extensive working hours [2]. Moreover, the emergence of ride-hailing services (e.g., app-based transportation platforms) has significantly reduced the number of passengers using traditional taxi services and led to a drastic decline in the value of taxi licenses [3]. In some countries, it has also been reported that taxi and bus drivers lack access to pension contributions, medical coverage, and union representation [4]. Their income often depends on the number of trips completed and is subject to vehicle maintenance costs, resulting in high variability in earnings based on the type and condition of their vehicles [4]. Therefore, the study of health-related quality of life (QoL) among taxi workers becomes essential.

QoL is a multidimensional concept that encompasses various physical, psychological, and social well-being factors that vary according to geographic location and local working conditions [5]. Evidence suggests that taxi drivers experience higher rates of depression, anxiety, stress, and psychological distress than the general population [6]. Consequently, this occupational group should be prioritized in the development and implementation of targeted prevention strategies [6]. Furthermore, their physical health is often compromised due to the extended hours spent and the sedentary lifestyle associated with the profession, both of which contribute to the development of musculoskeletal problems [7].

QoL is not limited only to the absence of disease but implies the ability to enjoy a full and satisfying life [8]. One of the factors with the greatest impact on QoL is pain triggered mainly by musculoskeletal disorders (MSDs), which are defined as any injury or disorder that causes pain, inflammation, and degeneration in the musculoskeletal system and neurovascular structures [9]. MSDs constitute a global public health issue as they not only impair individual quality of life (QoL) but are also associated with significant morbidity and disability and substantial economic costs [10]. MSDs are among the leading causes of pain, physical disability, and work limitations worldwide. These conditions often result in reduced mobility, chronic discomfort, and functional impairment, which in turn negatively affect various domains of QoL, including physical, emotional, and social well-being [11]. Therefore, examining MSDs in relation to QoL can help identify populations at risk of poorer health outcomes, such as taxi drivers, and inform interventions aimed at improving overall well-being and functional capacity. Key risk factors for developing MSD include sedentary behaviour, awkward postures, exposure to whole body vibration, heavy lifting, manual material handling, repetitive activities, workload, poor diet, and psychosocial stressors [12]. Taxi drivers have been identified as having a high prevalence of lower back pain, knee pain, hypertension, gastrointestinal disorders, fatigue, and MSD compared to the general population [13]. Furthermore, the prevalence of lower back pain in taxi drivers ranges from 27.9% and 58% depending on the study population [10],[14]–[17]. Consistently, a recent systematic review and meta-analysis including 11 studies and 5277 taxi drivers reported a high overall prevalence of MSDs, with the lower back being the most frequently affected region, followed by the neck, shoulder, and knee [18].

In this context, the prevalence of MSD in the occupational setting of professional drivers in Chile and Spain, and its association with QoL, is unknown to date, despite this occupational group being widely recognized as susceptible to developing such conditions. This gap is particularly relevant given that existing literature on other professional sectors has demonstrated a direct association between QoL and work-related MSDs, specific to each occupation [19]. Therefore, the aim of the present study is to assess the prevalence of MSD and their association with QoL among taxi drivers in Valparaíso (Chile) and Zaragoza (Spain), taking into account relevant sociodemographic variables.

## 2. Materials and methods

### 2.1. Design

This study is part of the research project “Study on the State and Determinants of Physical and Mental Health of Taxi Drivers in Zaragoza (Spain) and Valparaíso (Chile)”. It is an observational study involving 217 active taxi drivers, 115 from Zaragoza and 102 from Valparaíso, aiming to explore how social, environmental, and occupational factors impact their physical and mental health. Zaragoza, with a population of around 700,000 and a strong service and logistics economy, offers a structured European context with a continental climate. In contrast, Valparaíso, a coastal Chilean city with about 300,000 inhabitants, presents a Latin American setting characterized by socioeconomic variability, dense traffic, and steep geography, all of which may influence drivers' well-being differently.

Participants were recruited using a mix of convenience and snowball sampling, ensuring a range of ages, work experiences, and genders. Data collection took place over a full year (Oct 2022–Nov 2023) to account for seasonal variations. Inclusion criteria required participants to be actively working as taxi drivers in either city with at least one year of professional experience. Individuals with current work incapacity were excluded. Although the achieved sample sizes in both cities exceeded the a priori minimum required to estimate the prevalence of MSDs with acceptable precision, the use of non-probability (convenience and snowball) sampling may still limit the generalizability of our findings to all taxi drivers in Valparaíso and Zaragoza. Details on the a priori sample size calculation and precision parameters are provided below.

### 2.2. Sample size

Because the primary objective of this cross-sectional study was to estimate the prevalence of MSDs in taxi drivers rather than to test a specific hypothesis, the sample size was determined using a precision-based approach for a single proportion instead of a formal power analysis. The required sample size (*n*) for each country was calculated using the standard formula for estimating a single proportion: *n* = *Z*² × *p*(1 − *p*) / *d²*, where Z corresponds to the standard normal deviate for a 95% confidence level (*Z* = 1.96), p is the expected prevalence of MSDs, and d is the desired margin of error (*d* = 0.10). The sample was determined using the MSD variable for Chilean workers, 49.8% [20]. The sample was calculated with a 95% confidence interval and a 10% margin of error. In addition, the sample size was increased by 5% to account for possible dropouts. The minimum sample size was 101. For Spain [21], with a prevalence of 40.26, the minimum sample size is 97. The sample was calculated with a 95% confidence interval and a 10% margin of error. In addition, the sample size was increased by 5% to account for possible dropouts. The minimum sample size was 97.

### 2.3. Ethics approval

This study was approved by the Research Ethics Committee of the Autonomous Community of Aragón (PI22–382) and by the Bioethics Committee of the Pontificia Universidad Católica de Valparaíso (BIOEPUCV–H 633–2023). All participants gave their voluntary consent by signing the informed consent form after being fully informed about the objectives of the study. They were also informed of their right to withdraw from the study at any time without consequences.

### 2.4. Instruments

#### 2.4.1. Sociodemographic and occupational profile

Sociodemographic data were recorded for each participant, considering the following information: age, gender, marital status, number of children, educational level, perceived financial situation, work shifts (daytime, mixed, and nocturnal), hours worked per day, years of work as a driver, and whether the individual had another job.

#### 2.4.2. Quality of life

The SF-36 [22] was used to assess QoL. The survey consists of 36 health status perception questions with Likert-type scales. The questions are grouped into 8 dimensions: physical function, physical role, bodily pain, general health, vitality, social function, emotional role, and mental health, which were transformed into a 0–100 metric. Subscales were then aggregated into the Physical Component Summary (PCS) and the Mental Component Summary (MCS) using the instrument's weighting and norm-based scoring (NBS), which rescales scores to *T*-scores with mean = 50 and *SD* = 10 based on general-population reference norms described in the SF-36 scoring manuals [23]. Under NBS, 50 represents the population average; values <50 indicate below-average health status, and values ≥50 indicate average or better status.

#### 2.4.3. Musculoskeletal disorders

The Standardized Nordic Questionnaire [24] was used to assess MSD. The instrument provides information on the prevalence of pain across 15 body regions: neck, right shoulder, left shoulder, right elbow/forearm, left elbow/forearm, right wrist/hand, left wrist/hand, dorsal spine, lumbar spine, right hip/leg, left hip/leg, right knee, left knee, right ankle/foot, and left ankle/foot. The first part of the questionnaire inquires about the presence and duration of pain in each of these areas. If pain is reported, respondents proceed to the second section, which characterizes the discomfort based on the presence or absence of pain in the past 12 months.

The instrument relies on dichotomous (yes/no) responses and a Likert scale format, minimizing reporting bias and inaccuracies regarding pain intensity and the duration of symptoms in affected regions.

### 2.5. Statistical analysis

Data analysis was performed with the Stata/MP 16.0 (StataCorp LLC, College Station, TX, USA) program for Windows. The statistical description was performed using mean with standard deviation (*M* ± *SD*) for continuous variables and frequencies with percentages for categorical variables (*n*, %). Comparisons between countries (Chile *vs*. Spain) were made for sociodemographic variables and QoL. Prevalences of MSD were presented as frequency and percentage (*n*, %) according to presence in the previous 12 months in taxi drivers. Participants with the highest number of painful regions were assessed by grouping them at the 50th percentile (MSD ≥ 2 regions), a definition commonly used in occupational and population studies. This choice balances clinical interpretability and model parsimony; we acknowledge that alternative thresholds exist [25]. To assess QoL, scores were recorded considering a cutoff value of 50, where scores above 50 indicate better QoL and scores below 50 indicate worse QoL [23]. QoL on each scale was compared between those drivers with two or more painful regions (MSD ≥ 2 regions). For continuous variables, distribution was assessed using the Shapiro–Wilk normality test. When the normality assumption was met (*p* ≥ 0.05), comparisons between groups were performed using Student's *t*-test; otherwise, the Mann–Whitney U test was applied. Categorical variables were compared using Pearson's *χ²* test, or Fisher's exact test when expected cell counts were <5. Finally, logistic regressions were performed to analyze the association between the most prevalent cases of MSD ≥ 2 regions with the PCS and MCS summary measures of QoL, adjusted for sociodemographic characteristics. Significant variables resulting from the univariate analyses were incorporated into the multivariate model for each country. Confounding gender and age variables were also incorporated in all models. As a result, predictors that did not show evidence of association in the univariate analyses for a given country did not enter the final multivariable model for that country. The goodness of fit of each logistic regression model was proven with the Hosmer–Lemeshow test.

## 3. Results

Table 1 shows the sociodemographic characteristics of the total sample analyzed by country. A significant association was observed between country and the following categories: marital status, number of children, education, perception of economic situation, working hours, and daily working hours.

**Table 1.** Sociodemographic characteristics.

	**Total (*n* = 217) (%)**	**Chile (*n* = 102) (%)**	**Spain (*n* = 115) (%)**	***P*-value**
**Age**	51.2 ± 11.1	53.2 ± 13.2	49.47 ± 8.6	**0.005**
**Gender**				
Male	193 (88.9)	94 (92.2)	99 (86.01)	0.155
Female	24 (11.1)	8 (7.8)	16 (13.9)	
**Civil status**				
Single	60 (27.7)	38 (37.3)	22 (19.1)	**<0.001**
In a relationship	8 (3.7)	2 (1.7)	6 (5.2)	
Married	119 (54.8)	41 (40.2)	78 (67.8)	
Divorced	30 (13.8)	21 (20.6)	9 (7.8)	
**No. of children**	1.8 ± 1.6	2.4 ± 2.0	1.21 ± 0.9	**<0.001**
**Education**				
Primary	33 (15.4)	10 (9.8)	23 (20.5)	**<0.001**
Secondary	74 (34.6)	58 (56.9)	16 (14.3)	
Post-secondary	97 (45.3)	24 (23.5)	73 (65.2)	
**Perception of financial status**				
Satisfied	140 (64.5)	51 (50.0)	89 (77.4)	**<0.001**
Unsatisfied	77 (35.5)	51 (50.0)	26 (22.6)	
**Work shifts**				
Daytime	147 (67.7)	93 (91.2)	54 (47.0)	**<0.001**
Mixed	59 (27.2)	0 (0.0)	59 (51.3)	
Nocturnal	11 (5.1)	9 (8.8)	2 (1.7)	
**Hours of work per day**				
<8 hours	29 (13.4)	21 (20.6)	8 (7.0)	**0.004**
≥8 hours	186 (85.7)	79 (77.5)	107 (93.0)	
**Years worked as a taxi driver**	16.4 ± 11.2	16.1 ± 12.3	16.5 ± 10.2	0.396
**Other profession**				
Yes	34 (15.7)	10 (40.0)	14 (18.4)	**0.028**
No	182 (84.3)	15 (60.0)	62 (81.6)	

Note: Data were expressed as mean ± standard deviation or mean (percentage).

**Figure 1.**
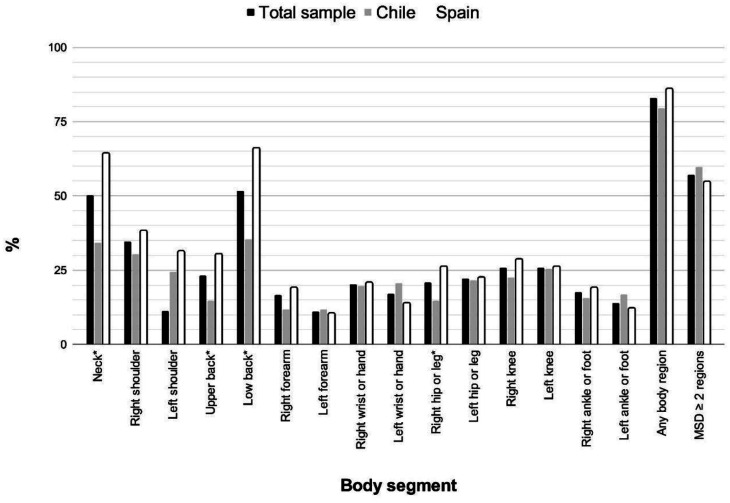
Prevalence of MSD among taxi drivers in Chile (grey), Spain (white), and the total sample (black) (MSD ≥ 2 regions. MSD, musculoskeletal disorder. * *p* < 0.05).

As shown in Figure 1 and Supplementary Table S1, 83% of the drivers felt pain in at least one area of the body during the last 12 months. The three areas with the highest prevalence of pain in the last 12 months were the lower back (51.6%), neck (50.2%), and right shoulder (34.6%). Differences between countries' body segment responses emerged in the neck (34.3% *vs*. 64.4%), upper back (14.7% Chile *vs*. 30.4% Spain), lower back (35.3% Chile *vs*. 66.1% Spain), and right hip or leg (14.7% Chile *vs*. 26.1% Spain).

Table 2 shows the sociodemographic characteristics of the groups (above *vs*. below 50 percentile) of the physical (PCS) and mental (MCS) components, in taxi drivers from Chile and Spain. Chilean drivers' PCS presented significant differences by age, where older age was related to worse PCS. Spanish drivers also presented a significant association between gender and PCS, with women having a worse PCS compared to men.

Among Spanish drivers, a significant association was found between having an additional occupation and better physical health, as measured by the PCS. Notably, however, only 13% of those with the highest PCS scores reported holding another job besides taxi driving. In both Chile and Spain, drivers reporting MSD ≥ 2 regions exhibited significantly poorer physical health.

Finally, in both countries, a perceived satisfactory financial situation was significantly associated with a better quality of life in both physical and mental dimensions. This relationship was especially pronounced in Spain, where the gap in QoL scores between those satisfied and dissatisfied with their financial situation was more substantial and consistent across both SF-36 domains.

Table 3 presents the results of a binary logistic regression model evaluating the association between QoL measured by the physical (PCS) and mental (MCS) components, and the presence of MSD ≥ 2 regions. The model was adjusted for sociodemographic variables in taxi drivers from Chile and Spain.

Both PCS and MCS showed significant associations with perceived financial situation. In Chile, drivers who reported dissatisfaction with their financial situation had a 2.8-fold increased risk of presenting a low PCS score. In contrast, among Spanish drivers, financial dissatisfaction was associated with a 3.1-fold increased risk of having a low MCS score. Additionally, increasing age was significantly associated with a higher risk of poor PCS scores among Chilean drivers.

Regarding MSDs, the presence of pain in two or more body regions (MSD ≥ 2 regions) was significantly associated with lower PCS scores in both countries (*p* < 0.001). However, no significant association was found between MSDs and MCS scores in either the Chilean or Spanish driver groups.

**Table 2.** Number of participants with decreased (<*T*-Score) or improved (≥*T*-Score) QoL values according to sociodemographic characteristics and MSD in Chilean and Spanish taxi drivers.

Variable	PCS-Chile		*p*	MCS-Chile		*p*	PCS-Spain		*p*	MCS-Spain		*p*
	≥*T*-Score	<*T*-Score		≥*T*-Score	<*T*-Score		≥*T*-Score	<*T*-Score		≥*T*-Score	<*T*-Score	
*Age (years)*	50.2 ± 13.2	56.5 ± 12.5	**0.015**	53.5 ± 13.1	52.5 ± 13.4	0.721	48.5 ± 8.9	50.9 ± 8.0	0.143	51.0 ± 8.1	48.3 ± 8.9	0.091
*Gender*												
Male	48 (90.6)	46 (93.9)	0.403	69 (94.5)	25 (86.2)	0.158	63 (91.3)	36 (78.3)	**0.048**	46 (92.0)	53 (81.5)	0.089
Female	5 (9.4)	3 (6.1)		4 (5.5)	4 (13.8)		6 (8.7)	10 (21.7)		4 (8.0)	12 (18.5)	
*Civil status*												
Single	20 (37.7)	18 (36.7)	0.635	25 (34.3)	13 (44.8)	0.105	12 (17.4)	10 (21.7)	0.228	11 (22.0)	11 (16.9)	0.529
In a relationship	0 (0.0)	2 (4.1)		0 (0.0)	2 (6.9)		5 (7.3)	1 (2.2)		2 (4.0)	4 (6.2)	
Married	21 (39.6)	20 (40.8)		31 (42.5)	10 (34.5)		49 (71.0)	29 (63.0)		35 (70.0)	43 (66.2)	
Divorced	12 (22.6)	9 (18.4)		17 (23.3)	4 (13.8)		3 (4.4)	6 (13.1)		2 (4.0)	7 (10.8)	
*Number of children*	1.9 ± 1.3	2.9 ± 2.4		2.3 ± 2.0	2.7 ± 1.8		1.2 ± 0.9	1.3 ± 1.0		1.2 ± 1.0	1.3 ± 0.9	
*Education*												
Primary	6 (11.3)	4 (8.2)	0.865	6 (8.2)	4 (13.8)	0.247	13 (19.1)	10 (22.7)	0.793	10 (20.4)	13 (20.6)	0.853
Secondary	28 (52.8)	30 (61.2)		45 (61.6)	13 (44.8)		9 (13.2)	7 (15.9)		6 (12.2)	10 (15.9)	
Post-secondary	13 (24.5)	11 (22.5)		14 (19.2)	10 (34.5)		46 (67.7)	27 (61.4)		33 (67.4)	40 (63.5)	
*Perception of financial status*												
Satisfied	32 (60.4)	19 (38.8)	**0.029**	41 (56.2)	10 (34.5)	**0.048**	59 (85.5)	30 (65.2)	**0.011**	44 (88.0)	45 (69.2)	**0.017**
Unsatisfied	21 (39.6)	30 (61.2)		32 (43.8)	19 (65.5)		10 (14.5)	16 (34.8)		6 (12.0)	20 (30.8)	
*Work shifts*												
Daytime	50 (94.3)	43 (87.8)	0.206	66 (90.4)	27 (93.1)	0.501	34 (49.4)	20 (43.5)	0.481	28 (56.0)	26 (40.0)	0.119
Mixed							33 (47.8)	26 (56.5)		22 (44.0)	37 (56.9)	
Nighttime	3 (5.7)	6 (12.2)		7 (9.6)	2 (6.9)		2 (2.9)	0 (0.0)		0 (0.0)	2 (3.1)	
*Hours of work per day*												
<8 hours	13 (24.5)	9 (18.4)	0.453	15 (20.6)	7 (24.1)	0.250	4 (5.8)	4 (8.7)	0.404	4 (8.0)	4 (6.2)	0.488
≥8 hours	40 (75.5)	39 (79.6)		58 (79.5)	21 (72.4)		65 (94.2)	42 (91.3)		46 (92.0)	61 (93.9)	
*Years worked as a taxi driver*	14.3 ± 11.4	18.1 ± 13.1	0.117	16.5 ± 12.6	15.2 ± 11.7	0.632	15.8 ± 9.3	17.7 ±11.3	0.324	16.5 ± 9.5	16.6 ± 10.7	0.946
*Other profession*												
Yes	13 (24.5)	11 (22.9)	0.849	19 (26.0)	5 (17.9)	0.278	9 (13.0)	1 (2.2)	**0.040**	3 (6.0)	7 (10.8)	0.290
No	40 (75.5)	37 (77.1)		54 (74.0)	23 (82.1)		60 (87.0)	45 (97.8)		47 (94.0)	58 (89.2)	
*MSD*												
<2 regions	30 (56.6)	11 (22.5)	**<0.001**	33 (45.2)	8 (27.6)	0.102	41 (59.4)	11 (23.9)	**<0.001**	24 (48.0)	28 (43.1)	0.599
≥2 regions	23 (43.4)	38 (77.6)		40 (54.8)	21 (72.4)		28 (40.6)	35 (74.1)		26 (52.0)	37 (56.9)	

Note: Number of participants (percentage); PCS: Physical Component Summary; MCS: Mental Component Summary; MSD: musculoskeletal disorder. *T*-Score: A score above 50 indicates a good QoL perception, while scores below 50 indicate a poor QoL perception.

**Table 3.** Logistic regression for the association between PCS and MCS of QoL with MSD ≥ 2 regions, adjusted for sociodemographic characteristics in taxi drivers.

		PCS (<*T*-Score)-Chile	P-value	MSC (<*T*-Score)-Chile	P-value	PCS (<*T*-Score)-Spain	P-value	MSC (<*T*-Score)-Spain	*P*-value
		*OR* [95% *CI*]^a^		*OR* [95% *CI*]		*OR* [95% *CI*]		*OR* [95% *CI*]	
MSD	<2 regions	1 Ref.		1 Ref.		1 Ref.		1 Ref.	
	≥2 regions	5.80 [2.20–15.27]	**<0.001**	1.89 [0.72–4.95]	0.198	3.39 [1.39–8.22]	**0.007**	0.99 [0.44–2.24]	0.976
Perception of financial status	Satisfied	1 Ref.		1 Ref.		1 Ref.		1 Ref.	
	Unsatisfied	2.83 [1.14–7.04]	**0.026**	2.23 [0.91–5.63]	0.078	2.21 [0.82–5.99]	0.117	3.10 [1.08–8.84]	**0.035**
Other profession	No	1 Ref.		1 Ref.		1 Ref.		1 Ref.	
	Yes	-	-	-	-	3.90 [0.45–34.03]	0.218	0.53 [0.12–2.31]	0.399
Age (years)		1.05 [1.01–1.09]	**0.010**	0.99 [0.96–1.04]	0.955	1.03 [0.98–1.08]	0.239	0.97 [0.92–1.01]	0.168
Gender	Female	1 Ref.		1 Ref.		1 Ref.		1 Ref.	
	Male	2.33 [0.45–12.23]	0.316	0.47 [0.99–2.21]	0.338	0.57 [0.17–1.94]	0.368	0.49 [0.14–1.75]	0.270
Hosmer–Lemeshow test^b^		0.196		0.283		0.633		0.199	

Note: a *OR:* odds ratios [confidence interval]; b A value above 0.05 indicates that the model fits the data; PCS: Physical Component Summary; MCS: Mental Component Summary; MSD: musculoskeletal disorder. *T*-Score: A score above 50 indicates a good QoL perception, while scores below 50 indicate a poor QoL perception. (–) In the Chilean sample, “other profession” did not show a statistically significant association with PCS or MCS in the univariate analyses and, according to the pre-specified variable-selection strategy, was not included in the multivariable models. Therefore, odds ratios for this variable are only reported for Zaragoza (Spain).

## 4. Discussion

The aim of this study was to examine the association of MSD with the QoL of Chilean and Spanish taxi drivers in the Valparaíso Region and the city of Zaragoza, considering the drivers' sociodemographic characteristics. The results indicate that over 80% of the drivers presented pain in at least one body region, with the lumbar spine, neck, and right shoulder being the most frequently affected areas in both countries. These findings suggest that such ailments may represent occupational health issues inherent to the profession, as noted in previous studies [15],[26].

The area with the highest prevalence of pain in the present study was the lower back. The prevalence among Chilean drivers (35.3%) was lower than that reported in studies from Japan (46%) and China (54%), while Spanish drivers exhibited a higher prevalence (66.1%) than both Asian counterparts [17],[27]. These findings underscore the potential impact of lower back pain on occupational performance in both groups. A Japanese study estimated that such pain in taxi drivers was significantly related to seat suitability, vehicle mileage, road vibration level, work stress, and time spent driving [17]. In line with these results, a national survey in Spain showed that diagnosed chronic low back pain affects 22% of the adult population and is associated with worse levels of physical and mental health, more comorbidities, and higher consumption of analgesics [28]. Factors that aggravate this condition include low educational level, occupational stress, and lack of social support, which coincide with the characteristics observed in the drivers analyzed. These parallels between the general population and taxi drivers highlight the need for specific interventions for this group. In this context, a personalized intervention combining progressive walking and pain education, delivered through physiotherapist-led sessions, has been shown to significantly reduce the recurrence of low back pain. The randomized clinical trial WalkBack demonstrated that this strategy not only extended the time to relapse (208 days *vs*. 112 in the control group) but also improved quality of life, reduced low back pain–related disability, and showed a high likelihood of being cost-effective from a societal perspective [29].

Neck (50.2%) and shoulder pain (36.4%) prevalence was higher than that reported in Ghana (neck: 15.2%, shoulder: 11%) but lower than in China (neck: 56.8%, shoulder: 43.2%). Evidence indicates that neck pain in drivers rarely occurs in isolation. Rather, it is commonly associated with concomitant musculoskeletal complaints in adjacent regions, particularly the shoulders and upper back. This is likely due to shared muscular overload in the upper body quadrant and prolonged postural demands during driving. A study conducted in Israel found that drivers with neck pain were eight times more likely to report shoulder pain and nearly six times more likely to report upper back pain, compared to those without neck pain [30].

Significant differences were also observed in the hip and right leg, suggesting that the right side of the body may be more affected by MSDs due to repetitive actions such as gear shifting, accelerating, and braking, especially in urban traffic conditions [14],[31]. Moreover, to maintain a stable posture, drivers hold static muscle tension for a prolonged period in the muscles of the neck, back, shoulders, and arms. This posture produces localized muscle fatigue leading to the development of muscle soreness [12], which meshes with the prevalence of MSD obtained in this study in the neck, shoulder, and back regions.

Taxi drivers differ from other professional drivers regarding risk factors for MSD in the lumbar region. This could be due to the fact that, 1) they spend more time behind the wheel (even over 10 hours a day as revealed by our results), 2) they are obliged to maintain stable driving postures that lead to postural stresses on the lumbar spine, and 3) they are confronted with various factors in the macro work environment (e.g., air pollutants, violence, and psychological stresses) that could increase work stress with the subsequent development of lower back pain [16]. From a practice standpoint, this pattern suggests actionable levers: capping continuous driving time, scheduling protected micro-breaks, promoting posture variability and brief in-vehicle exercises, and prioritizing seat/steering adjustability (including lumbar support) in fleet procurement and maintenance.

In relation to QoL, the results obtained reveal a poor QoL associated with PCS in both countries. The low QoL around drivers' physical component could be mainly associated with the fact that it is a static job and one of the jobs with the highest rate of sedentary lifestyle [32]. Despite some flexibility in scheduling, drivers often work long hours due to low wages and the highly competitive nature of the job [33]. Prolonged sitting and extended work hours contribute to a sedentary lifestyle, a major risk factor for chronic conditions such as diabetes, cardiovascular diseases, and obesity, and are associated with increased mortality [34]. The association between MSD ≥ 2 regions and lower PCS suggests a greater overall symptom load. Having pain in several body areas can limit sitting tolerance and everyday tasks (e.g., lifting, transfers), reduce activity and conditioning, and increase analgesic use, all of which lower perceived physical health. Psychosocial stressors (time pressure, traffic density, safety concerns) and sleep disruption from irregular schedules may further heighten pain and fatigue, with possible effects on mental well-being [35]–[37].

It should be noted that 92.16% of the sample analyzed in the present investigation were men. This finding aligns with international trends. For instance, a study conducted in China reported that 93.4% of taxi drivers were men [38], and the same trend is repeated in various studies conducted in South America. In a work conducted in Peru on MSD in taxi drivers, 83.7% of the respondents were male [39], while in a study conducted in Brazil on taxi drivers, for which sex was not an exclusion criterion, 100% of the respondents were male [40]. Therefore, although the data obtained does not represent a novelty for the group of interest, it is relevant to consider the masculinization of this work, as it could explain the trend of the results collected.

Our results indicate that drivers experiencing pain in more than two body regions show a significant association with lower QoL perceptions in the physical dimension. This finding is consistent with previous literature, which suggests that MSDs, due to their painful and chronic nature, are associated with worse QoL, primarily affecting the physical domain and, to a lesser extent, the mental dimension [11],[41]–[43]. The results obtained are also similar to those recorded in other occupational groups with high prevalence of MSD, such as teachers [44],[45], healthcare workers in charge of patient transport, and hospital cleaning and hygiene workers [46]. Regarding the Chilean drivers' age, it was observed that the age variable was significantly associated with a lower QoL perception in the PCS. This aligns with the results reported by Roux et al., who, in a cohort study of previously healthy individuals, concluded that QoL scores across multiple dimensions tend to decline with age [11]. Beyond the differences reported in the previous study, a higher MSD burden with advancing age is plausible via multiple pathways: cumulative exposure to driving-related mechanical load, age-related changes in discs, tendons, and cartilage, sarcopenia with reduced neuromuscular control, slower tissue repair, and greater comorbidity that may sensitize pain pathways. In professional drivers, these processes can interact with prolonged sitting, constrained postures, and repetitive pedal work, yielding more pain and functional limitation with age [47],[48].

Finally, the present investigation indicated that the perception of financial situation was significantly associated with the physical QoL dimension in Chilean drivers and with the MCS in Spanish drivers. This result coincides with reports from industrialized countries in Western Europe and North America that have described that lower socioeconomic status correlated with worse health [49]. These findings are particularly relevant in the context of taxi driving, as highlighted in studies from the United States, where drivers—especially those who do not own their vehicles—tend to work longer hours and face heightened stress due to financial insecurity. After deducting job-related expenses, many earn less than $5 per hour, a situation that compromises both public safety and the physical and mental health of drivers [6],[13]. Financial strain may plausibly lengthen shifts, compress recovery time, and heighten psychosocial stress, indirectly worsening both pain experience and QoL—an implication consistent with our country-specific patterns and relevant for policy.

The present study has several limitations. On the one hand, given that the study is cross-sectional in nature, the explanatory pathways analyzed generate hypotheses rather than causality, and the existence of residual confounding factors cannot be ruled out. It should also be noted that longitudinal studies will be necessary in the future to follow up on variables related to the presence of MSD in taxi drivers. On the other hand, although the questionnaires used to measure QoL and MSD are validated and widely used in research, they are based on participants' subjective perceptions. Consequently, QoL scores reflect self-reported health status over a limited recall window and may be affected by measurement error, reporting bias, and transient fluctuations in mood or context, which could attenuate or distort some associations. Moreover, MSD estimates were obtained via self-report over a 12-month recall window, which may introduce recall bias and misclassification; such bias could influence prevalence estimates and may vary across subgroups or between countries. Similarly, the sample size is not representative of all taxi drivers, so the results' generalizability to other regions may be limited. In addition, our operationalization of multi-site musculoskeletal pain as MSD ≥ 2 regions, while clinically interpretable and pre-specified, remains an arbitrary dichotomization that entails information loss relative to modeling the full count of painful regions; this choice may reduce statistical power and obscure potential dose–response patterns. Furthermore, some odds ratios in our multivariable logistic regression models showed wide 95% confidence intervals, indicating limited precision of these estimates. This is likely related to the relatively small number of events in some categories and the sparse distribution of certain covariates, which may lead to inflated odds ratio estimates and unstable confidence intervals in logistic regression models [50].

Future studies with larger samples should consider analyzing the count of painful sites to capture the gradient of pain burden more precisely.

## 5. Conclusions

A high MSD prevalence was identified, with 83% of the participants experiencing pain in some body region. The most affected segments were the neck, right shoulder, and lower back. Poor QoL related to physical health was observed as well.

Finally, we report a significant association between self-perceived financial status and QoL, which is consistent with the literature and suggests that the perception of socioeconomic status plays a fundamental role in workers' perceived health and well-being. Future studies should address not only the physical problems but also the psychological and emotional aspects of drivers' QoL.

The findings emphasize the need to pursue public health strategies and policies adapted to the particular working and sociodemographic conditions of this population. The information gathered in this study can support future research and help design interventions aimed at preventing the development of MSD, ultimately improving the QoL and overall well-being of taxi drivers in Chile and Spain. In this regard, a key proposal is the integration of health professionals, such as physiotherapists and occupational therapists, who can play a pivotal role in pain mitigation, functional rehabilitation, and ergonomic workplace adaptation. Additionally, the implementation of psychoeducational programs focused on occupational ergonomics is recommended, along with the inclusion of guided physical activity routines and the promotion of healthy habits, such as walking, active breaks, and stretching exercises.

## Data availability statement

Participants in this study did not provide written consent for their individual-level survey data to be shared in a public repository. Given the sensitive nature of the information collected on health status and working conditions, and the relatively small size of this occupational group, which may increase the risk of indirect identification, the full dataset cannot be made openly available. However, anonymised data may be made available from the corresponding author upon reasonable request for research purposes, in accordance with ethical and legal requirements and subject to additional approval by the relevant ethics committees if required.

## Use of AI tools declaration

The authors confirm that no Generative AI tools were used in the writing, editing, or content creation of this manuscript.


