# Supplementary material for: Association between musculoskeletal disorders and health-related quality of life in Chilean and Spanish taxi drivers
Source: AIMS Public Health. 2026 Jan 7;13(1):63–81. doi: 10.3934/publichealth.2026005 (PMC13084369; doi:10.3934/publichealth.2026005)
Supplement: Supplementary file 1 [file publichealth-13-01-005-s001.pdf]

Public Health

AIMS Public Health, 13(1): 63–81.
DOI: 10.3934/publichealth.2026005
Received: 29 August 2025
Revised: 17 November 2025
Accepted: 11 December 2025
Published: 07 January 2026

https://www.aimspress.com/journal/aimsph

Research article

Association between musculoskeletal disorders and health-related quality of life in Chilean and Spanish taxi drivers

Marta Marín-Berges[1], Alejandro Gómez-Bruton[2,3], Pablo A. Lizana[4,5,*], Maximiliano Cáceres[4], Pía Magaña[4] and Isabel Iguacel[2,6,7]

1 IBiOPS, Instituto de Investigación Sanitaria Aragón, Universidad de Zaragoza, Zaragoza, Spain
2 Centro de Investigación Biomédica en Red de Fisiopatología de la Obesidad y Nutrición (CIBEROBN), Instituto de Salud Carlos III, Madrid, Spain
3 EXER-GENUD (Growth, Exercise, Nutrition and Development), Faculty of Health and Sport Sciences, Huesca, University of Zaragoza, Spain
4 Laboratory of Epidemiology and Morphological Sciences, Instituto de Biología, Pontificia Universidad Católica de Valparaíso, Valparaíso 2373223, Chile
5 Center for Interdisciplinary Research in Biomedicine, Biotechnology and Well-Being (CID3B), Pontificia Universidad Católica de Valparaíso, Valparaíso, Chile
6 Instituto Agroalimentario de Aragón (IA2), Zaragoza, Spain
7 NUTRI-GENUD (Growth, Exercise, Nutrition and Development), Faculty of Health Sciences, Zaragoza, University of Zaragoza, Spain

* **Correspondence:** Email: pablo.lizana@pucv.cl; Tel: +5699433911.

Supplementary

Table S1. Prevalence of MSD in taxi drivers in Chile and Spain.

	Prevalence 12M *n* (%)			*p*-value
	Total sample	Chile	Spain	
Neck	109 (50.2)	35 (34.3)	74 (64.4)	**<0.001**
Right shoulder	75 (34.6)	31 (30.4)	44 (38.3)	0.224
Left shoulder	61 (28.1)	25 (24.5)	36 (31.3)	0.266
Upper back	50 (23.0)	15 (14.7)	35 (30.4)	**0.006**
Low back	112 (51.6)	36 (35.3)	76 (66.1)	**<0.001**
Right forearm	34 (15.7)	12 (11.8)	22 (19.1)	0.136
Left forearm	24 (11.1)	12 (11.8)	12 (10.4)	0.755
Right wrist or hand	44 (20.3)	20 (19.6)	24 (20.9)	0.818
Left wrist or hand	37 (17.1)	21 (20.6)	16 (13.9)	0.192
Right hip or leg	45 (20.7)	15 (14.7)	30 (26.1)	**0.039**
Left hip or leg	48 (22.1)	22 (21.6)	26 (22.6)	0.854
Right knee	56 (25.8)	23 (22.6)	33 (28.7)	0.302
Left knee	56 (25.8)	26 (25.5)	30 (26.1)	0.920
Right ankle or foot	38 (17.5)	16 (15.7)	22 (19.1)	0.505
Left ankle or foot	30 (13.8)	16 (15.7)	14 (12.2)	0.454
Any body region	180 (83.0)	81 (79.4)	99 (86.1)	0.192
MSD ≥ p50[a]	124 (57.1)	61 (59.8)	63 (54.8)	0.456

Note: a MSD ≥ 2 regions. MSD, Musculoskeletal disorder.

AIMS Press

© 2026 the Author(s), licensee AIMS Press. This is an open access article distributed under the terms of the Creative Commons Attribution License (https://creativecommons.org/licenses/by/4.0)
